# Who can claim the ownership to the blueprints of my body parts?

**DOI:** 10.1002/cre2.187

**Published:** 2019-04-25

**Authors:** Asbjørn Jokstad

**Affiliations:** ^1^ Faculty of Health Sciences, UiT The Arctic University of Norway Tromsø Norway

Advances in computer‐assisted design and manufacturing (CAD‐CAM) technologies enable the fabrication of individualised biomimetic body parts for an increasing range of damaged tissues and organs. CAD‐CAM in dentistry evolved 30 years ago for making intracoronal ceramic restorations, whereas the predominating CAD‐CAM–based devices today are intraoral crowns and prostheses that have been milled, printed, or sintered. The turnover of new technologies is rapid and diversified and pushed by hardware and software innovations, lower prices, and the seamless exchange of data facilitated by open data file formats (Jokstad, 2017). We can expect that newer and better dental biomaterials that are suitable for CAM will emerge as a response to the need for better clinical performance than current materials on the market. The good news is that if there is a need to replace an existing intraoral device, one may resend the original blueprint of the intraoral device, or a modification thereof, to any production device. The bad news is that the blueprint of the intraoral device can be anywhere and nowhere.

Technically, the word “blueprint” is no longer used and imply a CAD file, of which perhaps .stl, .amf, and .obj data file formats are the three most common in dentistry, amongst a portfolio of different subtractive and additive manufacturing file formats.

Apart from replacing existing intraoral devices with newer and better materials, one can also envisage needs such as replacing a CAD‐CAM–based single crown with a crack or breakage, a denture with a broken flange, or a fixed prosthesis showing an opaque zirconia core because of delamination of the veneering ceramic. True, everything may be remade from scratch, but the task is quite cumbersome and time‐consuming because of the need for removing the damaged prosthesis, make new impressions and a maxilla‐mandibular registration, and fabricate temporary solutions. Alternatively, it is feasible today, given that the doctor can access the blueprint of the existing intraoral device, to click on a computer that uploads the blueprint for inspection and modification on the screen and an additional click can start a subtractive or additive production device somewhere. Another scenario is that a patient retains a blueprint of his or her intraoral device and can negotiate costs with alternative dental care providers, albeit recognising that some traditionalist providers will likely disprove and object to such initiatives.

Despite having monitored the development of CAD‐CAM in dentistry, I fail to recall any papers clarifying or discussing who is the actual owner of the CAD file of the intraoral devices placed in patients' mouth. It is not evident whether the owner of this blueprint is the patient, the doctor, the designer of the intraoral device, the CAD software company, the production device company, or the owner of the production device; or does the ownership of the blueprint belong to the payer, who may perhaps be a third party? A further complication is that in many countries, the legal responsibility is placed firmly on the doctor for assuring an appropriate design of the intraoral device, including the choice of biomaterials and their handling, whereas the production device today may be operated and located in a different country and applicable to their respective national law. Hence, CAD files today can be anywhere and nowhere, although still subjected to governmental patient privacy regulations in all different countries involved. Incidentally, one may also argue that the designer of the intraoral device, for example, a dental technician, has an intellectual property right to the CAD design represented in the data file.

Because CAD‐CAM intraoral devices are becoming increasingly common, and we know that these devices will need to be replaced eventually, there is an urgent need to establish best practices and protocols, including a clarification of the blueprint ownership. The doctor is legally responsible for what enters the mouths of patients, so it seems prudent that at least a copy of the blueprint of the intraoral device is retained in the patient records for documentation. It also seems prudent that doctors refrain from giving carte blanche to the designer of the intraoral device or to the production device centre to proceed with refabricating an intraoral device from an old blueprint before the doctor has provided input or approved this blueprint.

Two overviews about regulatory aspects and legal considerations relative to CAD‐CAM–based intraoral devices do not specifically address blueprint ownership (Montmartin et al., 2015; Otero, Vijverman, & Mommaerts, 2017). The first paper contains a statement alluding to proprietorship, but its interpretation is ambiguous, that is, “Any medical device is likely to be protected by one or more patents so that it is not reproduced. The nonrespect of this intellectual property leads to the production of counterfeits, punishable by law.” The Council of European Dentists issued a statement about medical devices regulation and chairside CAD‐CAM procedures recently, but do not address blueprint ownership (CED, Council of European Dentists, 2018). The analogue focus in papers found in the orthopedic or surgery literature also does not address the blueprint ownership (Morrison et al., 2015). Admittedly, discussing the ownership of the blueprints for CAD‐CAM–based body parts that may have been designed by use of finite element analyses, subjected to a particular post‐processing treatment, and implanted during a complicated surgical operation in a hospital is different from discussing ownership of the blueprints to replaceable dental devices.

The real contentious debates revolve about CAD‐CAM–based devices that include live cells enabled by bioprinting, and particularly if these live cells do not originate from the individual receiving the device (Harbaugh, 2015). Bioprinting in context to dentistry is currently only limited to cranio‐oro‐maxillofacial plastic surgery, and the regulatory frameworks in Europe and the United States appear very complex, including the question about ownership of both the blueprint as well as the actual device (Hourd, Medcalf, Segal, & Williams, 2015). The potential future use and misuse of bioprinting have prompted many bioethicists and medical experts to voice concerns about ethical aspects such as human enhancement, rejuvenation medicine, unclear safety and risks, patent rights, and excessive costs and distributive justice (de Vries, Oerlemans, Trommelmans, Dierickx, & Gordijn, 2008). More recently, the issues of ownership of CAD‐CAM–based body parts has been raised, although more in the titles of articles rather than in the article contents (Gilbert, O'Connell, Mladenovska, & Dodds, 2018; Li, 2014; Vermeulen, Haddow, Seymour, Faulkner‐Jones, & Shu, 2017). The higher interest in body parts ownership is likely a reflection of the phenomenal advances that have been made lately in tissue engineering, combined with high monetary stakes. The most ambitious projects within this field of bioprinting aim to achieve full organogenesis, allegedly obliged by a global lack of enough donor organs (Ravnic et al., 2017). If successful, the societal impact will depend on whether the developers and future patent owners will operate for profit or perhaps less so.
